# Impact of Reduced-Dose Nonvitamin K Antagonist Oral Anticoagulants on Outcomes Compared to Warfarin in Korean Patients with Atrial Fibrillation: A Nationwide Population-Based Study

**DOI:** 10.3390/jcm10173918

**Published:** 2021-08-30

**Authors:** Sola Han, Young-Hoon Kim, Myung-Yong Lee, Oh Young Bang, Sung-Won Jang, Seongwook Han, Yoo-Jung Park, Seongsik Kang, Young Keun On, Hae Sun Suh

**Affiliations:** 1 Pharmaceutical Economics, Big Data Analysis & Policy, College of Pharmacy, Kyung Hee University, Seoul 02447, Korea; sola.han@khu.ac.kr; 2 Division of Cardiology, Department of Internal Medicine, Korea University, Seoul 02841, Korea; yhkmd@korea.ac.kr; 3 Division of Cardiology, Department of Internal Medicine, Dankook University, Cheonan 31116, Korea; mel_lee@dankook.ac.kr; 4 Department of Neurology, Samsung Medical Center, Sungkyunkwan University School of Medicine, Seoul 06355, Korea; ohyoung.bang@samsung.com; 5 Divison of Cardiology, Department of Internal Medicine, Catholic University of Korea, Seoul 06591, Korea; clement@naver.com; 6 Division of Cardiology, Department of Internal Medicine, Dongsan Hospital, Keimyung University School of Medicine, Daegu 42601, Korea; swhan@dsmc.or.kr; 7 Pfizer Korea Ltd., Seoul 04631, Korea; yoo-jung.park@pfizer.com (Y.-J.P.); seong-sik.kang@pfizer.com (S.K.); 8 Department of Cardiology, Samsung Medical Center, Sungkyunkwan University School of Medicine, Seoul 06355, Korea

**Keywords:** anticoagulants, atrial fibrillation, NOAC, stroke, systemic embolism, warfarin

## Abstract

Reduced-dose nonvitamin K antagonist oral anticoagulants (NOACs) are commonly prescribed to Asian patients with nonvalvular atrial fibrillation (NVAF). We aimed to compare the risk of stroke/systemic embolism (S/SE) and major bleeding (MB) between patients treated with reduced-dose NOACs and those treated with warfarin, using the claims database in Korea. Patients with NVAF newly initiated on oral anticoagulants (OACs; apixaban, dabigatran, rivaroxaban, and warfarin) between 1 July 2015 and 30 November 2016 were included. Among all patients with NVAF treated with OACs, 5249, 6033, 7602, and 8648 patients were treated with reduced-dose apixaban, dabigatran, rivaroxaban, and warfarin, respectively. Patients treated with reduced-dose NOACs were older and had higher CHA_2_DS_2_-VASc and HAS-BLED scores than those treated with warfarin. Compared to warfarin, all reduced-dose NOACs showed significantly lower risk of S/SE (hazard ratios (95% confidence interval), 0.63 (0.52–0.75) for apixaban; 0.51 (0.42–0.61) for dabigatran; and 0.67 (0.57–0.79) for rivaroxaban) and MB (0.54 (0.45–0.65) for apixaban; 0.58 (0.49–0.69) for dabigatran; 0.73 (0.63–0.85) for rivaroxaban). In the real-world practice among Asians with NVAF, all reduced-dose NOACs were associated with a significantly lower risk of S/SE and MB compared to those of warfarin.

## 1. Introduction

Oral anticoagulants (OACs) have taken a crucial role in the treatment strategy of atrial fibrillation (AF) to prevent stroke [1,2,3]. Although warfarin is known as an effective agent for reducing the risk of stroke in AF, there have been challenges in using warfarin in Asian patients, including the possibility of difference in the optimal target of international normalized ratio (INR) [4,5,6]. Several studies investigating acceptable outcomes with lower INR levels in Asian populations have considered not only bleeding or ischemic events but also its pharmacokinetic/pharmacodynamic characteristics [7,8,9,10]. Moreover, due to the higher incidence rate of hemorrhagic stroke in Asians undergoing Vitamin K Antagonist (VKA) therapy, careful assessment of bleeding complications of OACs among Asians in general is crucial [6,11].

Nonvitamin K OACs (NOACs), now recommended as the choice of treatment, are widely used based on evidence from recent large randomized controlled trials (RCTs), which have demonstrated their noninferiority and superior efficacy/tolerability compared with those of warfarin [12,13,14,15]. All these studies have shown fewer bleeding events with a standard dose of NOACs compared to warfarin. Nevertheless, it has been reported that physicians tend to prescribe reduced-dose NOACs to Asian patients with AF in real practice, owing to a major concern of bleeding complications [16,17]. The real-world evaluations assessing the effectiveness of reduced-dose NOACs in Asian are limited, and only a few studies reported about the conflicting results in the view of the stroke event risk [18,19].

Therefore, this study aimed to compare the risk of stroke/systemic embolism (S/SE) and major bleeding (MB) in Korean patients treated with reduced-dose NOACs versus warfarin under real clinical practice.

## 2. Materials and Methods

### 2.1. Data Source

The Korean Health Insurance Review and Assessment (HIRA) Service claims data from 1 January 2007 to 30 November 2016 were used in this study. The HIRA database includes data covering the entire population under the universal health insurance system in Korea [20]. These data included information on demographics, diagnoses, health services, and prescriptions and were provided after being anonymized [20]. Diagnosis codes in these data were based on the International Classification of Diseases 10th Revision (ICD-10) [20]. The Pusan National University Institutional Review Board determined that this study did not require ethical review (PNU IRB/2016_137_HR).

### 2.2. Study Population

The study included OAC-naïve patients who had one or more prescriptions for apixaban, dabigatran, or rivaroxaban during the intake period of 1 July 2015–30 November 2016. We defined the index date as the first OAC prescription date. The inclusion criteria were patients above 18 years of age on the index date and a minimum of two outpatient visits or one hospitalization with AF diagnosis (i.e., ICD-10 code I48) before or on the index date. The latter inclusion criterion performed well in a previous validation study with a positive predictive value of 94.1% [21]. As the Korean health insurance policy allows reimbursement for anticoagulant prescription in AF patients with CHA_2_DS_2_-VASc score of 2 or higher, all included patients are considered indicated for anticoagulation therapy.

Patients with the following conditions were excluded: hip or knee replacement surgery within six weeks prior to or on the index date; valvular AF, prosthetic heart valves, venous thromboembolism, thyrotoxicosis, hypertrophic cardiomyopathy, end-stage chronic kidney disease, kidney transplant, dialysis, pericarditis, elective defibrillation, radiofrequency ablation, or left atrial appendage occlusion during the 12-month baseline period; NOAC or warfarin use within 1 year prior to the index date; more than one OAC prescription on the index date; both standard and reduced dose on the index date; both standard and other dose on the index date; or both reduced and other dose on the index date.

Standard dose was defined as the general recommended dose for patients with AF as specified in the package insert (i.e., 10, 300, and 20 mg as a daily dose for apixaban, dabigatran, and rivaroxaban, respectively). Reduced dose was defined as the recommended dose for patients who have renal dysfunction and/or low body weight or are elderly as specified in the package insert (i.e., 5, 220, and 15 mg as a daily dose for apixaban, dabigatran, and rivaroxaban, respectively). Other dose was defined as a dose lower than the reduced dose on label or higher than the standard dose (Table 1).

The follow-up period was from the index date to the date of switching from index OAC treatment to another OAC, treatment discontinuation, death, or 30 November 2016, whichever came first. Discontinuation was defined as the absence of prescription for any OACs within 30 days after the last day of supply of the last filled prescription. Medication switch was defined as the presence of a prescription filled for nonindex OAC treatment within 30 days after the last day of supply of the last filled prescription.

### 2.3. Study Endpoints

S/SE was the effectiveness outcome and included ischemic stroke, hemorrhagic stroke, and systemic embolism. Diagnosis codes with hospitalization and brain computed tomography (CT) or magnetic resonance imaging (MRI) records were used to identify stroke [22,23], whereas diagnosis codes with hospitalization and any CT or MRI records were used to identify SE. MB was the safety outcome and included intracranial hemorrhage (ICH), gastrointestinal (GI) bleeding, and other bleeding. ICH was defined using diagnosis codes with hospitalization and brain CT or MRI records. The remaining safety outcomes (i.e., GI and other bleeding) were defined using diagnosis codes with hospitalization. Primary and all secondary diagnosis codes were used to define each outcome. Additional details regarding the effectiveness and safety outcomes are provided in Supplementary Materials Table S1.

### 2.4. Statistical Analysis

Propensity score matching (PSM) using propensity scores was conducted to balance baseline demographics and clinical characteristics. Propensity score is the probability of receiving treatment, which is based on the observed characteristics [24]. We used PSM rather than inverse probability of treatment weighting (IPTW) because the proportion of patients who received treatment contrary to prediction was substantial, which can cause unstable results. If there is instability in the estimated propensity score, PSM may be preferable to IPTW [24]. Propensity scores were estimated using logistic regression and included information on baseline demographics and clinical characteristics, including age, sex, insurance type, baseline medication use, baseline comorbidities, Charlson Comorbidity Index (CCI), CHA_2_DS_2_-VASc score, HAS-BLED score, and individual risk factors of CHA_2_DS_2_-VASc and HAS-BLED scores. The details on CHA_2_DS_2_-VASc and HAS-BLED scores, baseline medication use, and CCI are provided in Supplementary Materials Tables S2–S5. For the matching to make comparable groups, we used a caliper of 0.01. The balance for each variable between the treatment groups was evaluated using standardized differences in the matched sample [24], and a standardized difference below 10% was considered acceptable [24]. In the presence of an imbalance, the variable was included in the Cox proportional hazards model. The Cox proportional hazards model was used to compare outcomes between the treatment groups. Proportional hazard assumption was evaluated using several methods, such as log–log plots, Schoenfeld’s residuals, and time-dependent covariates [25]. If the proportional hazard assumption was not met, hazard ratio (HR) with 95% confidence interval (CI) at 1 year was estimated using the extended Cox model which contains the product term of the variable with a log of time [25]. 

For analysis, the following three comparisons were performed: (1) reduced-dose apixaban versus warfarin, (2) reduced-dose dabigatran versus warfarin, and (3) reduced-dose rivaroxaban versus warfarin. All statistical analyses were performed using SAS 9.4 (SAS Institute Inc., Cary, NC, USA).

## 3. Results

### 3.1. Baseline Characteristics

**Figure 1:**
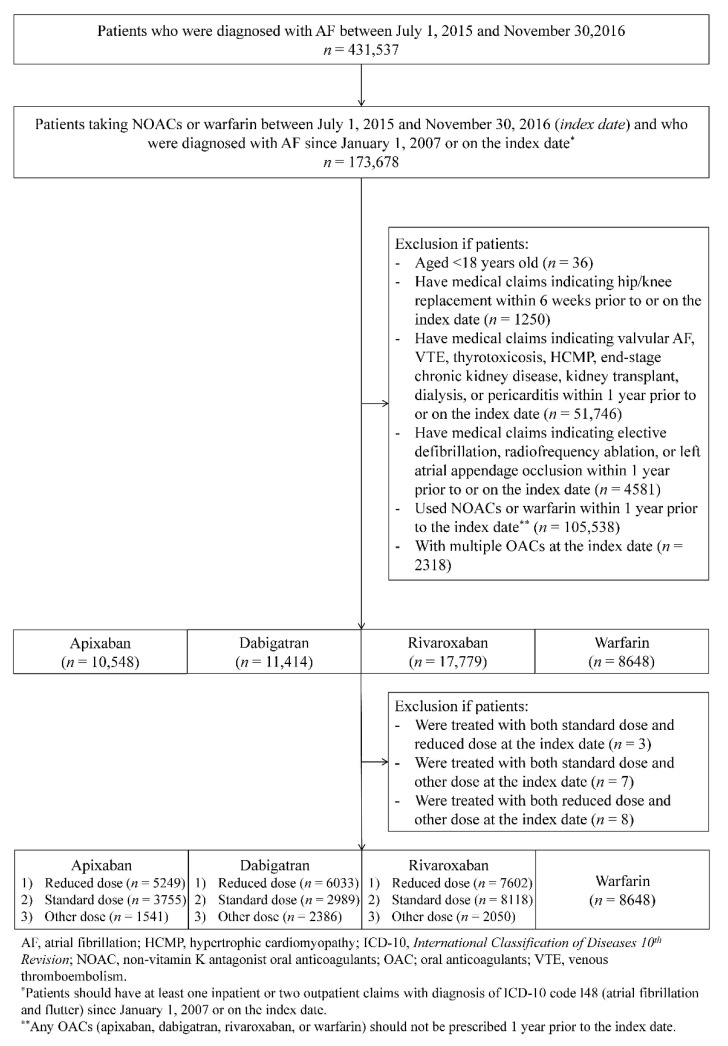
Cohort creation flow of oral anticoagulant users.

A total of 48,389 OAC-naïve patients who newly initiated OACs were identified: 10,548 apixaban, 11,414 dabigatran, 17,779 rivaroxaban, and 8648 warfarin. Among these patients, 5249, 6033, and 7602 patients received reduced-dose apixaban, dabigatran, and rivaroxaban, respectively (Figure 1). Before matching, patients treated with reduced-dose NOACs were older and had higher CHA_2_DS_2_-VASc and HAS-BLED scores compared to those of patients treated with warfarin (Supplementary Materials Table S6). After matching, all differences in baseline characteristics were balanced (*p* > 0.05; absolute standardized difference <0.1; Table 2).

### 3.2. Effectiveness Outcomes

**Figure 2:**
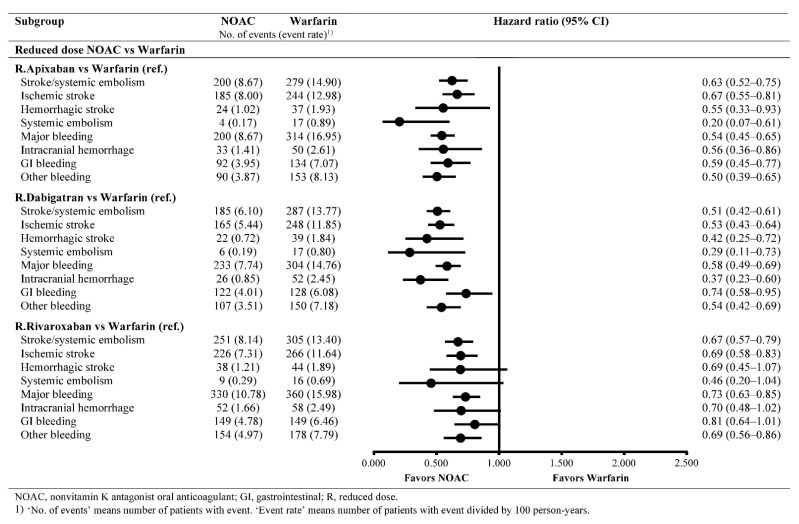
Event rates and hazard ratios in propensity score-matched cohorts for the comparison of reduced-dose NOACs with warfarin.

The effectiveness outcomes based on the comparisons of reduced-dose NOACs with warfarin are summarized in Figure 2. Reduced-dose apixaban, dabigatran, and rivaroxaban were associated with a lower risk of S/SE compared to that of warfarin (HR (95% CI), 0.63 (0.52–0.75) for reduced-dose apixaban versus warfarin; HR (95% CI), 0.51 (0.42–0.61) for reduced-dose dabigatran versus warfarin; HR (95% CI), 0.67 (0.57–0.79) for reduced-dose rivaroxaban versus warfarin) (Figure 2). Among the comparisons of reduced-dose NOACs with warfarin, dabigatran showed the lowest crude event rate of S/SE (5.88 per 100 person-years) (Table 3).

### 3.3. Safety Outcomes

The safety outcomes based on the comparisons of reduced-dose NOACs with warfarin are summarized in Figure 2. Reduced-dose apixaban, dabigatran, and rivaroxaban were associated with a lower risk of MB compared with that of warfarin (HR (95% CI), 0.54 (0.45–0.65) for reduced-dose apixaban versus warfarin; HR (95% CI), 0.58 (0.49–0.69) for reduced-dose dabigatran versus warfarin; and HR (95% CI), 0.73 (0.63–0.85) for reduced-dose rivaroxaban versus warfarin) (Figure 2). Among the comparisons of reduced-dose NOACs with warfarin, dabigatran showed the lowest crude event rate of MB (7.60 per 100 person-years) (Table 3).

## 4. Discussion

In this study, we measured the risk of thromboembolic and bleeding events in patients with AF who were newly initiated on reduced-dose NOACs or warfarin for stroke prevention. This large real-world cohort of Korean patients taking daily doses of 5 mg apixaban, 15 mg rivaroxaban, or 220 mg dabigatran was compared with those taking warfarin. We found that the risk of S/SE was lower in patients treated with 5 mg apixaban, 15 mg rivaroxaban, or 220 mg dabigatran daily than in those treated with warfarin. Furthermore, significantly lower bleeding risk was observed in all reduced-dose NOAC treatment groups compared with the warfarin group.

We observed differences in the baseline characteristics between the reduced-dose NOAC and warfarin cohorts. There were more females in the NOAC cohorts; furthermore, the patients were older and had higher CHA_2_DS_2_-VASc scores with more comorbidities in general. Among the NOAC cohorts, the patients treated with dabigatran generally had a lower number of comorbid diseases, were younger, and had lower CHA_2_DS_2_-VASc and HAS-BLED scores than those treated with the other NOACs. On the other hand, the CHA_2_DS_2_-VASc score and proportion of patients with comorbidities, such as renal disease, history of bleeding, and prior ischemic stroke, were the highest among those treated with apixaban within the NOAC cohort. Similar patient characteristic patterns have also been observed in the Western population, wherein those with apixaban had more comorbid diseases and were generally older [18].

Dose reduction in NOACs is primarily recommended according to the dose-reduction criteria established by pivotal RCTs. In the 2019 AHA/ACC/HRS guidelines, dose adjustment of NOACs is based on the Food and Drug Administration dosing guidelines [26]. In patients for whom combination therapy with a P2Y12 inhibitor and NOAC is considered, reduced-dose rivaroxaban (15 mg once daily) is recommended [26]. The 2018 ACCP guidelines recommend label-adjusted NOAC dosing, which reflects the dose-reduction criteria of the pivotal RCTs [27]. This consensus on the use of NOACs is also followed by the 2021 EHRA practical guidelines [28]. However, in Japan, the standard rivaroxaban dose is 15 mg once daily, and 10 mg rivaroxaban once daily is prescribed for patients with renal impairment based on the pharmacokinetic data available for elderly Japanese patients with AF. In Korea, the dose recommendation for all NOACs follows US or EU therapeutic labels.

Each NOAC has specific criteria for dose reduction, which might be considered complex by physicians. Due to the concerns regarding excessive bleeding among Asian patients on warfarin therapy [4,29], maintenance of a lower INR range has become a usual treatment approach [7,9], which may deliver on the perception that lower doses of NOACs would be sufficient. Subsequently, choosing the right dose for each patient has become a challenge in clinical settings, and the assumption that a certain proportion of patients treated with NOACs did not receive appropriate doses cannot be ruled out in this study. 

In a US prospective registry for patients with AF receiving NOACs (*n* = 5738), 87% of patients were prescribed on-label NOAC doses; the rates of underdosing and overdosing were 9.4% and 3.4%, respectively [30]. These rates were markedly higher in the Asian population [31]. As mentioned in the Comparison Study of Drugs for Symptom Control and Complication Prevention of AF (CODE-AF) Registry with Korean patients with AF, substantial proportion of Korean patients with AF received off-label reduced-dose NOAC: 53.9% of rivaroxaban users, 55% of apixaban users, and 23.5% of edoxaban users [31]. The off-label reduced dose was administered to older patients and females, and the patients had lower body weight, renal dysfunction, history of stroke and bleeding, hypertension, and concomitant medication use.

There are several potential reasons for frequent use of reduced-dose NOACs in Asian patients with AF. Asians tended to have lower body weight and smaller body size than non-Asians who were mainly included in the pivotal NOAC RCTs. Moreover, Asians showed a higher risk of ICH when compared to non-Asians in the warfarin era [4]. The clinical factors such as previous bleeding history and fluctuation of renal function or body weight could influence the physicians’ choice of dosing in real-world practice. Thus, clinicians may prescribe OAC therapy in Asian AF patients in a conservative manner due to the possibility of adverse bleeding events.

Several subanalyses have revealed that outcomes after using the on-label reduced dose of NOACs versus warfarin are overall consistent with standard dose versus warfarin. In a meta-analysis of three pivotal RCTs, reduced-dose NOACs in patients who were eligible for dose reduction were associated with similar risk of ischemic stroke and lower risk of hemorrhagic stroke, ICH, and fatal bleeding compared to those of warfarin [32]. Favorable result of the reduced-dose NOACs in comparison to warfarin was also documented in a meta-analysis of 18 real-world data studies, especially in Asians [33]. Other studies have demonstrated that clinical benefits were reduced with NOAC underdosing; however, off-label reduced doses continue to be prescribed [31]. Large randomized or observational studies are warranted to determine the efficacy and safety of reduced-dose NOACs in real-world settings.

Although this study could not differentiate between the appropriate and inappropriate use of reduced-dose NOACs, the study findings revealed the clinically significant data on reduced-dose NOACs that may provide benefits in real-world practice compared to warfarin therapy. However, caution is warranted in its interpretation and use of reduced-dose NOACs should be strictly based on prescribing labels. This finding suggests that NOACs, regardless of dosage, may provide better clinical outcomes in patients with AF for whom warfarin is indicated. Poor INR control and low treatment satisfaction of VKA users among Korean patients with AF might be one of the potential underlying reasons for the observed differences in outcomes between warfarin and reduced-dose NOACs in this study [34]. In another study evaluating the effectiveness and safety of NOACs versus warfarin among the Korean population with AF, NOACs had an overall favorable clinical benefit compared to that of warfarin, although 50–75% of the cohort included patients on reduced-dose NOAC treatment [17]. Similar results were obtained from our previous study in which the risk of S/SE and MB were compared between NOACs (including all dosages) and warfarin in the OAC taking AF patients [35]. Considering that reduced dosing is recommended for patient groups associated with a higher risk of not only bleeding (HAS-BLED) but also for stroke (CHA_2_DS_2_-VASc), this study’s results provide additional information on the benefit of NOACs over warfarin in Asian patients requiring anticoagulation for stroke prevention.

### Limitations

This study has some inherent limitations associated with the retrospective analysis of claims data. First, the HIRA database does not include data on some patient characteristics such as body weight or laboratory data, including INR and those related to renal and liver function. Therefore, assessing whether patients were managed with appropriate doses of anticoagulants was challenging. In addition, the adequacy of anticoagulation in patients in the warfarin group could not be determined due to lack of information on INR, which may affect the clinical interpretation of the study results, as Asian AF patients tend to be kept at a lower INR range compared to that of non-Asians. Second, the claims database that we utilized in this study cannot discern the specific type of AF (i.e., paroxysmal, persistent). Third, despite the careful adjustment of confounders by PSM, thereby allowing a close balance between the groups, the presence of unmeasured confounding factors cannot be ruled out. Finally, the study follow-up period was relatively short as reimbursement of NOACs as first-line therapy was initiated on 1 July 2015. Therefore, further studies are necessary to explore the long-term effectiveness and safety of NOACs. However, the Cox proportional hazards model revealed significant differences in all comparisons; therefore, the relatively short follow-up period might not have had a significant impact on the main findings of this study.

## 5. Conclusions

In a large observational study of reduced-dose NOACs, NOACs were associated with lower thromboembolic and bleeding risk compared to those of warfarin. Nevertheless, the use of NOACs should adhere to dose-reduction criteria indicated in the therapeutic label. This study provides further evidence for the use of NOACs in Asian patients with NVAF.

## Figures and Tables

**Table 1:** Definition of dose groups at the index date.

Groups	Daily Doses (mg)		
	Apixaban	Dabigatran	Rivaroxaban
Standard dose ^1^	10	300	20
Reduced dose ^2^	5 *	220	15
Other dose ^3^	<5 or >10	<220 or >300	<15 or >20

^1^ Standard dose was defined as the general recommended dose for patients with atrial fibrillation as specified in the package insert. ^2^ Reduced dose was defined as the recommended dose for patients with renal dysfunction and/or low body weight and those who are elderly as specified in the package insert. ^3^ Other dose was defined as a dose lower than the reduced dose or higher than the standard dose indicated on the drug label. * 7.5 mg/day apixaban was considered as reduced dose.

**Table 2:** Baseline characteristics of patients receiving reduced-dose nonvitamin K antagonist oral anticoagulants and warfarin after propensity score matching.

	Propensity Score Matching					
	After ^1,2^					
	R.Apixaban (*n* = 4774)	Warfarin (*n* = 4774)	R.Dabigatran (*n* = 5221)	Warfarin (*n* = 5221)	R.Rivaroxaban (*n* = 5746)	Warfarin (*n* = 5746)
Age (years), mean	75.50	75.45	73.94	73.85	74.21	74.15
Female, %	49.37	49.73	44.38	44.86	45.04	45.41
CHA_2_DS_2_-VASc, mean	4.97	4.99	4.67	4.68	4.71	4.73
HAS-BLED, mean	3.74	3.75	3.63	3.63	3.66	3.67
CCI, mean	4.55	4.55	4.18	4.20	4.29	4.33
Insurance, %						
National health insurance	92.42	91.94	92.01	91.59	92.24	92.36
Medical aid	7.58	8.06	7.99	8.41	7.76	7.64
Medical history, %						
Heart failure	45.35	45.92	42.56	42.56	43.84	44.43
Hypertension	87.81	88.37	88.89	89.29	88.58	89.00
Diabetes	56.66	57.27	53.36	53.65	54.47	55.33
Ischemic stroke	38.04	38.77	34.09	34.17	34.08	34.16
Vascular disease	32.45	31.57	31.68	31.95	32.02	32.09
Renal disease (CKD3/4)	3.10	3.10	1.26	1.11	2.61	2.61
Bleeding	14.62	14.73	9.84	9.83	12.63	13.00
Medication history, %						
NSAIDs	80.23	79.10	79.95	80.52	79.24	79.38
Antiplatelets	75.12	75.05	74.28	74.33	75.20	75.18
Antiarrhythmics	48.66	47.72	46.75	46.56	45.70	45.49
Statins	58.59	59.70	56.22	57.04	54.72	55.46
PPI	46.19	45.60	43.33	43.04	44.33	43.63
H2RA	70.34	69.92	68.86	68.51	68.53	68.97
Digoxin	27.34	27.17	27.93	27.70	28.30	28.70

CCI, Charlson Comorbidity Index; CHA_2_DS_2_-VASc, congestive heart failure, hypertension, age ≥ 75 years, diabetes mellitus, stroke, vascular disease, age 65–74 years, and sex; CKD, chronic kidney disease; HAS-BLED, hypertension, abnormal renal and liver function, stroke, bleeding, labile international normalized ratio, elderly, drugs or alcohol; H2RA, H_2_-receptor antagonists; PPI, proton pump inhibitors; R, reduced dose. ^1^ *p* values were not significant for all comparisons (R.Apixaban vs. Warfarin; R.Dabigatran vs. Warfarin; R.Rivaroxaban vs. Warfarin). ^2^ Absolute standardized differences were not above 10% for all comparisons (R.Apixaban vs. Warfarin; R.Dabigatran vs. Warfarin; R.Rivaroxaban vs. Warfarin).

**Table 3:** The summary of crude event rates in patients taking reduced-dose NOACs and warfarin.

	Reduced Dosing			Warfarin (*n* = 8648)	R.Api (*n* = 4774)	Warfarin (*n* = 4774)	R.Dabi (*n* = 5221)	Warfarin (*n* = 5221)	R.Riva (*n* = 5746)	Warfarin (*n* = 5746)
	Apixaban (*n* = 5249)	Dabigatran (*n* = 6033)	Rivaroxaban (*n* = 7602)							
	Crude Event Rates ^1^				Event Rates in Matched Cohorts ^1^					
S/SE	8.56	5.88	7.30	11.86	8.67	14.90	6.10	13.77	8.14	13.40
IS	7.92	5.28	6.52	10.33	8.00	12.98	5.44	11.85	7.31	11.64
HS	0.97	0.64	1.04	1.52	1.02	1.93	0.72	1.84	1.21	1.89
SE	0.15	0.17	0.31	0.76	0.17	0.89	0.19	0.80	0.29	0.69
MB	8.40	7.60	9.94	13.53	8.67	16.95	7.74	14.76	10.78	15.98
ICH	1.35	0.81	1.47	2.06	1.41	2.61	0.85	2.45	1.66	2.49
GI	3.89	4.01	4.48	5.57	3.95	7.07	4.01	6.08	4.78	6.46
Oth	3.70	3.39	4.56	6.58	3.87	8.13	3.51	7.18	4.97	7.79

GI, gastrointestinal bleeding; HS, hemorrhagic stroke; ICH, intracranial hemorrhage; IS, ischemic stroke; MB, major bleeding; Oth, other bleeding; R.Api, reduced dose of apixaban; R.Dabi, reduced dose of dabigatran; R.Riva, reduced dose of rivaroxaban; S/SE, stroke/systemic embolism; SE, systemic embolism. ^1^ “Event rate” indicates the number of patients with event divided by 100 person-years.

## Data Availability

The study data were extracted and analyzed from the Korea Health Insurance Review & Assessment Service (HIRA) claims database, and additional data may be obtained from a third party (with appropriate authorization approval) but are not publicly available.

## Supplementary Materials

The following are available online at https://www.mdpi.com/article/10.3390/jcm10173918/s1, Table S1: International Classification of Disease, Tenth Revision (ICD-10) codes for the study outcomes, Table S2: Scoring and International Classification of Disease, Tenth Revision (ICD-10) codes for the factors included in the CHA_2_DS_2_-VASc score, Table S3: Scoring and International Classification of Disease, Tenth Revision (ICD-10) codes for the factors include in the HAS-BLED score, Table S4: Types of baseline medications, Table S5: Scoring and International Classification of Disease, Tenth Revision (ICD-10) codes for the factors included in the Charlson Comorbidity Index, Table S6: Baseline characteristics of patients receiving reduced-dose nonvitamin K antagonist oral anticoagulants and warfarin before propensity score matching.
