# Supplementary material for: Impact of Reduced-Dose Nonvitamin K Antagonist Oral Anticoagulants on Outcomes Compared to Warfarin in Korean Patients with Atrial Fibrillation: A Nationwide Population-Based Study
Source: J Clin Med. 2021 Aug 30;10(17):3918. doi: 10.3390/jcm10173918 (PMC8432037; doi:10.3390/jcm10173918)
Supplement: Supplementary file 1 [file jcm-10-03918-s001.zip › jcm-1359967-supplementary.pdf]

Table S1. *International Classification of Disease, Tenth Revision* (ICD-10) codes for the study outcomes.

**Outcomes**		**ICD-10 codes**
Stroke or systemic embolism	Ischemic stroke*	G45.9, I63, and I69.3
	Haemorrhagic stroke*	I60, I61, I62, I69.0, I69.1, and I69.2
	Systemic embolism**	I74
Major bleeding***	Intracranial hemorrhage*	I60, I61, I62, I69.0, I69.1, I69.2, S06.4, S06.5, S06.6, and S06.8
	Gastrointestinal bleeding	I85.0, I98.3, K22.11, K22.6, K22.8, K25.0, K25.2, K25.4, K25.6, K26.0, K26.2, K26.4, K26.6, K27.0, K27.2, K27.4, K27.6, K28.0, K28.2, K28.4, K28.6, K29.0, K31.81, K55.21, K62.5, K92.0, K92.1, and K92.2
	Bleeding from other sites	D62, H44.8, H35.72, H35.6, H31.3, H21.0, H11.3, H05.2, H47.0, H43.1, I31.2, N02.0–N02.9, N42.1, N83.1, N85.7, N92.0, N92.3, N93.0, N93.8, N93.9, M25.0, R23.3, R04.0, R04.1, R04.2, R04.8, R04.9, T79.2, T81.0, N95.0, R31.0, R31.1, R31.8, R58, T45.5, Y44.2, and D68.3

* Brain CT/MRI procedure codes and hospitalization were also required to define ischemic stroke, hemorrhagic stroke or intracranial hemorrhage

** Any CT/MRI procedure codes and hospitalization were also required to define systemic embolism

*** Hospitalization was also required to define major bleeding (i.e., intracranial hemorrhage, gastrointestinal bleeding and bleeding from other sites)

Table S2. Scoring and *International Classification of Disease, Tenth Revision* (ICD-10) codes for the factors included in the CHA_2DS_2-VASc score.

**Condition**	**ICD-10 codes**	**Point**
Congestive heart failure	I50	1
Hypertension	I10-I15	1
Age	≥75 years	2
Diabetes	E10–E14	1
Stroke	I63, I69.3, and G45.9	2
Vascular disease	I21, I25.2, and I70–I73	1
Age	65–74 years	1
Sex	Female	1

Table S3. Scoring and *International Classification of Disease, Tenth Revision* (ICD-10) codes for the factors include in the HAS-BLED score.

**Condition**	**ICD-10 codes**	**Point**
Hypertension	I10–I15	1
Abnormal renal disease	N18.3 and N18.4	1
Abnormal liver function	B15–B19, C22, D68.4, I98.2, I98.3, K70–K77, and Z94.4	1
Stroke	I63, I69.3, and G45.9	1
Bleeding history or predisposition*	I60, I61, I62, I69.0, I69.1, I69.2, S06.4, S06.5, S06.6, S06.8, I85.0, I98.3, K22.11, K22.6, K22.8, K25.0, K25.2, K25.4, K25.6, K26.0, K26.2, K26.4, K26.6, K27.0, K27.2, K27.4, K27.6, K28.0, K28.2, K28.4, K28.6, K29.0, K31.81, K55.21, K62.5, K92.0, K92.1, K92.2, D62, H44.8, H35.72, H35.6, H31.3, H21.0, H11.3, H05.2, H47.0, H43.1, I31.2, N02.0-N02.9, N42.1, N83.1, N85.7, N92.0, N92.3, N93.0, N93.8, N93.9, M25.0, R23.3, R04.0, R04.1, R04.2, R04.8, R04.9, T79.2, T81.0, N95.0, R31.0, R31.1, R31.8, R58, T45.5, Y44.2, and D68.3	1
Elderly	≥65 years	1
Drug therapy	Antiplatelets and NSAIDs	1
Alcoholism	E24.4, F10, G31.2, G62.1, G72.1, I42.6, K29.2, K70, K86.0, O35.4, P04.3, Q86.0, T51.0, X45, X65, Y15, Y90-Y91, Z50.2, Z71.4, and Z72.1	1

* A blood transfusion was also required to define a history of bleeding from other sites

Table S4. Types of baseline medications.

Class	Drugs
NSAIDs	Bromfenac, celecoxib, diclofenac, etodolac, fenoprofen, flurbiprofen, ibuprofen, indomethacin, ketoprofen, ketorolac, naproxen, meclofenamate, mefenamic acid, meloxicam, nabumetone, oxaprozin, piroxicam, sulindac, and tolmetin
Antiplatelets	Aspirin, clopidogrel, prasugrel, ticlopidine, cilostazol, abciximab, tirofiban, dipyridamole, and ticagrelor
Proton pump inhibitors	Omeprazole, pantoprazole, lansoprazole, rabeprazole, esomeprazole, and dexlansoprazole
H_2-receptor antagonists	Cimetidine, ranitidine, famotidine, nizatidine, roxatidine, and lafutidine
Antiarrhythmics	Quinidine, procainamide, mexiletine, propafenone, flecainide, amiodarone, bretylium, dronedarone, propranolol, atenolol, esmolol, verapamil, diltiazem, and sotalol
Digoxin	Digoxin
Statins	Atorvastatin, fluvastatin, lovastatin, pitavastatin, pravastatin, rosuvastatin, and simvastatin

NSAID, nonsteroidal antiinflammatory drug

Table S5. Scoring and *International Classification of Disease, Tenth Revision* (ICD-10) codes for the factors included in the Charlson Comorbidity Index.

Condition	ICD-10 codes	Point
Cerebrovascular disease	G45.x, G46.x, H34.0, and I60.x–I69.x	1
Congestive heart failure	I09.9, I11.0, I13.0, I13.2, I25.5, I42.0, I42.5-I42.9, I43.x, I50.x, and P29.0	1
Chronic pulmonary disease	I27.8, I27.9, J40.x–J47.x, J60.x–J67.x, J68.4, J70.1, and J70.3	1
Dementia	F00.x–F03.x, F05.1, G30.x, and G31.1	1
Diabetes without chronic complication	E10.0, E10.1, E10.6, E10.8, E10.9, E11.0, E11.1, E11.6, E11.8, E11.9, E12.0, E12.1, E12.6, E12.8, E12.9, E13.0, E13.1, E13.6, E13.8, E13.9, E14.0, E14.1, E14.6, E14.8, and E14.9	1
Mild liver disease	B18.x, K70.0-K70.3, K70.9, K71.3-K71.5, K71.7, K73.x, K74.x, K76.0, K76.2-K76.4, K76.8, K76.9, and Z94.4	1
Myocardial infarction	I21.x, I22.x, and I25.2	1
Peripheral vascular disease	I70.x, I71.x, I73.1, I73.8, I73.9, I77.1, I79.0, I79.2, K55.1, K55.8, K55.9, Z95.8, and Z95.9	1
Peptic ulcer disease	K25.x–K28.x,	1
Rheumatologic disease	M05.x, M06.x, M32.x–M34.x M31.5M35.1, M35.3, and M36.0	1
Diabetes with chronic complication	E10.2–E10.5, E10.7, E11.2–E11.5, E11.7, E12.2-E12.5, E12.7, E13.2–E13.5, E13.7, E14.2–E14.5, and E14.7	2
Hemiplegia or paraplegia	G04.1, G11.4, G80.1, G80.2, G81.x, G82.x, G83.0-G83.4, and G83.9	2
Any malignancy, including leukemia and lymphoma	C00.x–C26.x, C30.x–C34.x, C37.x–C41.x, C43.x, C45.x–C58.x, C60.x–C76.x, C81.x–C85.x, C88.x, and C90.x–C97.x	2
Renal disease	I12.0, I13.1, N03.2-N03.7, N05.2-N05.7, N18.x, N19.x, N25.0, Z49.0-Z49.2, Z94.0, and Z99.2	2
Moderate or severe liver disease	I85.0, I85.9, I86.4, I98.2, K70.4, K71.1, K72.1, K72.9, K76.5, K76.6, and K76.7	3
AIDS/HIV	B20.x–B22.x and B24.x	6
Metastatic solid tumor	C77.x–C80.x	6

Table S6. Baseline characteristics of patients receiving reduced-dose nonvitamin K antagonist oral anticoagulants and warfarin before propensity score matching.

	**Propensity score matching**					
	**Before**					
	**R.Apixaban (n=5249)**	**Warfarin (n=8648)**	**R.Dabigatran (n=6033)**	**Warfarin (n=8648)**	**R.Rivaroxaban (n=7602)**	**Warfarin (n=8648)**
Age (years), mean	76.02	68.73	74.33	68.73	75.25	68.73
Female, %	52.22	37.44	46.91	37.44	49.83	37.44
CHA_2DS_2-VASc, mean	5.04	4.06	4.68	4.06	4.80	4.06
HAS-BLED, mean	3.74	3.34	3.59	3.34	3.62	3.34
CCI, mean	4.53	4.04	4.01	4.04	4.18	4.04
Insurance, %						
National health insurance	92.55	92.45	91.63	92.45	92.50	92.45
Medical aid	7.45	7.55	8.37	7.55	7.50	7.55
Medical history, %						
Heart failure	46.47	41.00	42.91	41.00	46.70	41.00
Hypertension	88.59	80.80	90.37	80.80	91.12	80.80
Diabetes	57.13	51.57	52.35	51.57	54.74	51.57
Ischemic stroke	37.59	32.15	31.23	32.15	29.43	32.15
Vascular disease	31.22	29.24	30.33	29.24	30.72	29.24
Renal disease (CKD3/4)	2.97	2.45	1.09	2.45	2.10	2.45
Bleeding	14.00	13.54	8.52	13.54	10.16	13.54
Medication history, %						
NSAIDs	81.01	76.38	81.78	76.38	81.62	76.38
Antiplatelets	75.65	70.06	75.80	70.06	77.95	70.06
Antiarrhythmics	49.55	48.60	46.43	48.60	42.98	48.60
Statins	60.35	51.41	58.51	51.41	55.29	51.41
PPI	46.52	42.77	42.55	42.77	42.36	42.77
H2RA	70.58	66.27	68.41	66.27	67.73	66.27
Digoxin	26.16	27.56	26.65	27.56	27.35	27.56

CCI, Charlson Comorbidity Index; CHA_2DS_2-VASc, congestive heart failure, hypertension, age ≥ 75 years, diabetes mellitus, stroke, vascular disease, age 65–74 years, and sex; CKD, chronic kidney disease; HAS-BLED, hypertension, abnormal renal and liver function, stroke, bleeding, labile international normalized ratio, elderly, drugs or alcohol; H2RA, H_2-receptor antagonists; PPI, proton pump inhibitors; R, reduced dose.
